# Measuring Anxiety in Autistic Children: Assessing the Validity of the Anxiety Scale for Children with Autism Spectrum Disorder

**DOI:** 10.3390/ejihpe14090168

**Published:** 2024-09-20

**Authors:** Keira Goulding, Linda Campbell, Emily Freeman

**Affiliations:** 1 School of Psychological Sciences, The University of Newcastle, Callaghan, NSW 2308, Australia; keira.goulding@uon.edu.au (K.G.);; 2 Healthy Minds Research Program, Hunter Medical Research Institute, Newcastle, NSW 2308, Australia

**Keywords:** autism, anxiety, autistic children, anxiety scale for children with autism spectrum disorder

## Abstract

The present study assessed the validity of one of the first autism-specific anxiety measures, the Anxiety Scale for Children with Autism Spectrum Disorder (ASC-ASD), and compared its ability to predict parent-reported clinical anxiety to a ‘traditional’ anxiety measure, the Spence Children’s Anxiety Scale (SCAS). Whether the inclusion of the child form for each measure improved the predictive ability of the parent forms was also examined. Eighty-seven parents of autistic children, aged 8–12 years, completed the ASC-ASD, the SCAS, and the Social Communication Questionnaire (SCQ), a screener for autism characteristics. Of these parents, 56 had their child complete the ASC-ASD and SCAS. The children with a reported anxiety diagnosis were rated significantly higher by their parents on both the SCAS and the ASC-ASD compared to the non-anxious children. Pearson’s correlation coefficients indicated that the ASC-ASD had good divergent and convergent validity, as demonstrated by a poor, non-significant correlation with the SCQ and a strong, significant correlation with the SCAS. Regression analyses indicated that while the ASC-ASD was a significant predictor of parent-reported clinical anxiety in autistic children, the SCAS was not. Neither model was improved with the inclusion of the respective child form. This study is the first to demonstrate the ability of the ASC-ASD to predict child clinical anxiety disorder status and adds to the growing body of evidence for the validity of this measure. The findings also suggest that parent reports of anxiety may be sufficient to identify autistic children warranting further clinical investigation of anxiety in this age group.

## 1. Introduction

Anxiety disorders are one of the most commonly co-occurring conditions in children diagnosed with Autism Spectrum Disorder (ASD), with an estimated 39.6% of autistic children also meeting the criteria for at least one anxiety disorder [1]. Autistic children are also at an increased risk of experiencing ‘sub-clinical anxiety’, where their anxiety fails to meet the threshold for a clinical diagnosis, but still significantly interferes with their everyday life [2]. This interference has been reported to have far-reaching effects, impacting a child’s ability to function at school and their family and peer relationships [3], as well as decreasing the quality of life of both autistic children and their parents [4]. As autistic people are already at increased risk of experiencing poorer outcomes compared to their non-autistic peers [5], co-occurring anxiety may potentially exacerbate this risk. Consistent with this suggestion, other research has found that the presence of an anxiety disorder in autistic youth is associated with increased depression symptomology and self-injurious behaviours compared to youth with an ASD diagnosis alone [6].

Given the potential detrimental impacts, it is important to consider methods to accurately screen for anxiety disorders in autistic children to ensure the appropriate supports are put into place to minimise the negative consequences of anxiety symptomatology. Concerningly, Kerns et al. [7] suggested that the expression of anxiety in autistic individuals may not always be consistent with the symptoms of the anxiety disorders outlined in the *Diagnostic and Statistical Manual of Mental Disorders* (5th ed.; *DSM-5*) [8], despite causing significant impairments to daily functioning. Understanding the nature and expression of anxiety in autistic individuals is further complicated by the unique presentation of anxiety symptoms within this population, as these individuals can display both typical and atypical anxiety symptoms [9]. These atypical symptoms may include anxiety around changes to routine or exposure to new situations, people, or events; unusual specific fears; ritualistic behaviours; social fearfulness; or sensory-related anxieties [10]. The presence of atypical anxiety symptoms is a common occurrence in autistic individuals, with one study reporting that of the autistic youth found to have anxiety symptoms, 17% presented with traditional anxiety, 15% with atypical anxiety, and 31% with both [10]. Additionally, more severe social difficulties were found to be related to the presence of atypical symptoms, while language ability and hypersensitivity were predictive of typical or traditional anxiety [10].

While there has been some attempt to validate anxiety measures developed for non-autistic populations for use in autistic individuals, these ‘traditional’ measures often fail to account for the full range of anxiety expression within this population [9]. This varied expression may limit the validity and reliability of measures designed for use in non-autistic populations, and highlights the need for autism-specific assessments. Supporting this idea, Wigham and McConachie [11] demonstrated through their assessment of the psychometric properties of several traditional anxiety measures, only three could be considered ‘robust’. While their analysis revealed that the Spence Children’s Anxiety Scale (SCAS) [12], the Screen for Child Anxiety-Related Emotional Disorders (SCARED) [13], and the Revised Children’s Anxiety and Depression Scale (RCADS) [14] could be considered acceptable for autistic individuals, they suggested that these measures be used with caution, as they still fail to account for the broad range of anxiety expression found in autistic individuals. Similarly, a study by Lecavalier et al. [15] of 10 traditional anxiety measures reported that only 4 could be considered ‘appropriate with conditions’ for clinical trials in autistic children. Other researchers have also found that the underlying factor structure of traditional measures often differs between youth with and without an ASD diagnosis [16]. Problematically, this may result in a lack of understanding regarding the prevalence and expression of anxiety within this population, as well as potentially failing to identify targets for treatment or intervention. 

In response to the lack of autism-specific anxiety measures, the Anxiety Scale for Children with Autism Spectrum Disorder (ASC-ASD) [17] was created to better reflect the atypical and typical anxiety symptomology expressed by autistic children. Since its release, the ASC-ASD has been shown to be useful in measuring anxiety in autistic children, with children with parent-reported clinical anxiety scoring higher on the ASC-ASD than those with no such diagnosis [18]. It has also been demonstrated to have good convergent validity with the SCARED [17] and the Mini-International Neuropsychiatric Interview for Children and Adolescents [19], and was found to be correlated with the School Anxiety Scale-Teacher Report [20]. However, as it is a relatively new measure, it still requires further evidence for its validity and to determine whether it better captures the unique and varied presentation of anxiety commonly demonstrated by autistic children compared to existing traditional measures.

To date, only one study has assessed the convergent validity of the ASC-ASD with the SCAS, one of the most commonly used anxiety measures in autism research [21]. In that study, den Houting et al. [22] found that the total scores of the parent forms of the SCAS and the ASC-ASD were moderately to strongly correlated, while the total scores of the child forms for each measure were strongly correlated. Their study also found that while parents tended to endorse more items on the SCAS, children were more likely to endorse items on the ASC-ASD. Parent and child total scores for the SCAS were weakly to moderately correlated, while parent and child total scores for the ASC-ASD were moderately correlated. Neither of the anxiety measures scores were correlated with the autism screener, the Social Communication Questionnaire (SCQ) [23], suggesting that they measured anxiety rather than autism characteristics. While this research provided some evidence for the validity of the ASC-ASD and highlighted its potential clinical utility, it used a relatively small sample (*n* = 30 for the SCAS, *n* = 24 for the ASC-ASD) and did not explore the predictive ability of either measure. 

Despite the weak-to-moderate correlations observed between the parent–child pairs on the SCAS and ASC-ASD in den Houting et al.’s (22) study, and the low-to-moderate agreement between the parent–child dyads on the ASC-ASD in Soh et al.’s [19] study, other research into parent–child discrepancies in anxiety ratings amongst autistic children has yielded mixed results. While some studies have found moderate, or moderate-to-strong correlations between parent–child anxiety ratings [24,25], others have found poor agreement between parent–child pairs [26,27]. Various reasons have been suggested for the observed discrepancies, including a lack of self-insight on the part of the child into their own internal emotional state, differing perspectives on the experience of anxiety between the parent and child, or the hesitancy of the child to truthfully disclose the extent of their difficulties [28]. 

Other researchers have also implicated the impact of clinically significant anxiety on score discrepancies. For example, in a recent study by Kalvin et al. [29], comparing autistic youth with clinical anxiety, autistic youth without anxiety, and ‘typically developing’ controls, the parents of autistic children with anxiety rated their child’s anxiety significantly higher than the child did themselves, while the autistic youth without anxiety and the typically developing controls self-reported higher levels of anxiety compared to their parents. This finding suggests that autistic children with co-occurring anxiety disorders may be more likely to underreport their experience of anxiety compared to autistic children without clinically significant anxiety. 

The inconsistency in the findings also extends to the research investigating parent–child agreement with the SCAS. In one study by Magiati et al. [30] of 38 autistic youth in Singapore, the agreement between the reports of anxiety symptoms given by the children and their caregivers was rated overall as ‘moderately good’. This was particularly true for the subscales of the SCAS that assess generalized anxiety, separation anxiety, and physical injury fear symptoms; however, the researchers noted that stronger autism characteristics were related to poorer parent–child agreement. Interestingly, the mean parent ratings tended to be lower than the child ratings due to more generalized anxiety and obsessive–compulsive symptoms reported by autistic youth. This result is in contrast to previous research that observed that parents typically rate their child’s anxiety more severely than the child themselves [24,31]. 

Conversely, May, Cornish, and Rinehart [28] found poor agreement between autistic children and their parents when rating child anxiety on the SCAS, with only a weak significant correlation observed between parent and child scores on a subscale assessing separation anxiety symptoms. In contrast, significant associations were observed between the SCAS total score and nearly all its subscales for the non-autistic controls in this study, suggesting that the non-autistic parent–child pairs had greater levels of agreement than the autistic parent–child dyads. The parents of autistic children also reported significantly higher levels of child anxiety than the parents of non-autistic children, despite similar levels of anxiety reported by the autistic and non-autistic children. In total, these findings suggest that further exploration of the utility of both the parent and child forms of the SCAS in autistic children is warranted, as well as other anxiety measures that may better encapsulate the experience of anxiety for autistic children, such as the ASC-ASD. 

The current study aimed to replicate and expand on the work of den Houting et al. [22] by assessing the utility and validity of the ASC-ASD, as well as investigating whether the ASC-ASD or the SCAS is more appropriate for evaluating anxiety in autistic children. As the ASC-ASD was developed specifically for use in this population, it was hypothesised that while children with a reported anxiety disorder would score significantly higher on both the ASC-ASD and the SCAS than their non-anxious counterparts, a higher percentage of children would be correctly identified by the ASC-ASD compared to the SCAS. 

Based on the research examined, it was also predicted that the ASC-ASD would have good convergent validity with the SCAS and divergent validity with the SCQ, reflecting that the scale assesses anxiety symptoms rather than autism characteristics.

The present research also aimed to investigate whether the ASC-ASD or the SCAS was a better predictor of parent-reported clinically diagnosed anxiety in autistic children, and whether the predictive ability of the ASC-ASD and the SCAS was improved by the inclusion of the child form for each measure. Given that the scale was developed specifically to include items assessing autism-specific anxiety symptoms, it was expected that the ASC-ASD would be a superior predictor of parent-reported clinically diagnosed anxiety compared to the SCAS. Due to the observed discrepancies between parent–child anxiety ratings in the literature, it is unclear whether the child forms of the ASC-ASD or SCAS would improve the predictive ability of either measure; thus, no prediction was made regarding this.

## 2. Materials and Methods

### 2.1. Participants

One hundred twenty-seven parent participants self-selected to complete the online survey by responding to a recruitment advertisement on Facebook. The exclusion criteria included a parent-reported severe intellectual disability (i.e., IQ < 35) and no reported diagnosis of ASD. After exclusions, the final sample consisted of 87 parents (range = 20 to 53 years, M = 38.85, SD = 7.23) of children aged 8 to 12 years (M = 9.41, SD = 1.38) with a reported diagnosis of ASD. The participants who indicated that their child did not have an ASD diagnosis (*n* = 24), or who scored below the cut-off point on the SCQ (*n* = 16) were excluded prior to analysis. Thirty-four (39.1%) of the children were reported to have at least one anxiety disorder diagnosis, including generalized anxiety disorder, post-traumatic stress disorder, obsessive–compulsive disorder, or an unspecified anxiety disorder, which is consistent with the prevalence estimate specified by van Steensel, Bögels, and Perrin’s [1] meta-analysis.

Of the participating parents, 76 also had their child complete at least part of the survey, although 17 were excluded due to having no reported diagnosis of ASD, resulting in the inclusion of data from 59 children. Three of these children completed the SCAS only, and not the ASC-ASD. No reason was provided by the parents who did not have their children complete the survey, as child participation was optional for this study.

### 2.2. Demographic Characteristics

The parents completed a demographic questionnaire which asked questions about their child’s age, gender, age at diagnosis, school type, comorbidities, medications, and average hours of intervention or therapy. Questions about parental age, education, marital status, employment status, geographical region, and household income were also included. 

### 2.3. Autism Characteristics

The Social Communication Questionnaire—Lifetime Version (SCQ) [23] is a behavioural checklist used to screen for ASD in children aged over 4 years. The SCQ is completed by a parent or caregiver and consists of 40 yes/no items which assess the child’s communication skills and social functioning. While there are two versions of the SCQ, the present study used the Lifetime Version, which focuses on the child’s behaviour across their entire developmental history. A recent meta-analysis found that the Lifetime Version of the SCQ is an acceptable screener for ASD [32]. The first item in the SCQ is not scored and is used to determine whether the child can communicate verbally. If the child is non-verbal, the subsequent six items pertaining to language differences are omitted. For every other item in the SCQ, a point is given for each answer indicating atypical behaviour or development, and then summed to provide a total score. For verbal children, a maximum score of 39 is possible, while non-verbal children can score a maximum of 33. A higher score on the SCQ is indicative of the presence of a larger number of behaviours which are consistent with ASD characteristics. A cut-off point of 15 was used, with scores above this point used to confirm parent-reported ASD diagnosis. 

### 2.4. Anxiety

The Anxiety Scale for Children with Autism Spectrum Disorder (ASC-ASD) is the first anxiety measure specifically designed to assess both the typical and atypical symptoms of anxiety in autistic children. The items are drawn from the Revised Children’s Anxiety and Depression Scale (RCADS) and comprise four subscales: Separation Anxiety (SA), Uncertainty (U), Performance Anxiety (PA), and Anxious Arousal (AA). Both the parent (ASC-ASD-P) and child (ASC-ASD-C) forms of this measure consist of 24 items which are answered on a 4-point scale, where Never = 0, Sometimes = 1, Often = 2, and Always = 3. For the total score, the scores for each item are summed together, with a maximum possible score of 72. The subscale scores are calculated by summing the score for each item composing that domain. For both the ASC-ASD-C and ASC-ASD-P, a total score of ≥ 20 may reflect elevated levels of anxiety. Cronbach’s alpha indicated that the internal consistency was excellent for the total score of the parent form (α = 0.93) and the child form (α = 0.94). The internal consistency was good to excellent for all the subscales of the parent form (PA, α = 0.92; AA, α = 0.88; SA, α = 0.83; U, α = 0.90) and the child form (PA, α = 0.90; AA, α = 0.89; SA, α = 0.88; U, α = 0.89).

The Spence Children’s Anxiety Scale (SCAS) is a well-validated measure designed to assess anxiety in non-autistic populations. The parent form (SCAS-P) consists of 38 items which are scored to assess anxiety, as well as 1 open-ended unscored response. The child form (SCAS-C) consists of 38 anxiety items, 6 positive filler items, and 1 open-ended unscored response. The open-ended item asks about whether the child has any additional fears and, if so, how often they are impacted by these fears. The item responses on both forms are on a 4-point scale, where Never = 0, Sometimes = 1, Often = 2, and Always = 3. The total scores are calculated by summing the score for each anxiety item. The maximum possible score for both the parent and child form is 114. The scale is composed of six subscales: Panic Attack and Agoraphobia, Separation Anxiety, Physical Injury Fears, Social Phobia, Obsessive Compulsive, and Generalized Anxiety Disorder. The scores for each subscale are calculated by summing the scores of the items within that subscale. Both the total score and subscale scores are converted into *t*-scores, where a *t*-score of more than 60, or more than one standard deviation above the mean, is indicative of elevated anxiety warranting further clinical investigation. 

The internal consistency for the total score of the SCAS-P (α = 0.92) and SCAS-C (α = 0.89) was good to excellent, and good for the Social Phobia (SCAS-P, α = 0.82; SCAS-C, α = 0.87), Panic (SCAS-P, α = 0.84; SCAS-C, α = 0.85), Separation Anxiety (SCAS-P, α = 0.82; SCAS-C, α = 0.83), and Generalized Anxiety (SCAS-P, α = 0.82; Child, α = 0.80) subscales. The reliability was also acceptable for the SCAS-C Obsessive Compulsive subscale (α = 0.72). However, the SCAS-P Obsessive Compulsive subscale (α = 0.62), and the SCAS-P and SCAS-C Physical Injury subscales (Parent, α = 0.66; Child, α = 0.58) had poor-to-questionable internal consistency. No major improvements to Cronbach’s alpha were observed even with the removal of items from the subscales with less than good consistency. 

### 2.5. Procedure

The parents answered demographic questions before completing the SCQ, SCAS-P, and ASC-ASD-P as part of the online survey. The parents were then invited to allow their child to complete the SCAS-C and ASC-ASD-C. The parents were informed that they could assist their child with reading the questions or clarifying any of the items; however, the answers should be the child’s own. 

### 2.6. Data Analysis

Descriptive statistics were calculated for the demographic and study variables. The differences in the parent–child ratings and in the ratings for the anxiety measures for children with and without clinical anxiety were assessed using paired-samples *t*-tests. 

The number and percentage of participants whose reported anxiety disorder status was correctly identified by either the ASC-ASD or the SCAS was also calculated to further explore the clinical utility of these measures. 

The convergent and divergent validity were examined using Pearson’s correlation coefficients, which explored the strength and direction of the relationships between the ASC-ASD, SCAS, and the SCQ. 

As the dependent variable was categorical (diagnosed anxiety disorder = yes or no), sequential binomial logistic regression analyses were used to determine whether either anxiety measure was a significant predictor of parent-reported clinically diagnosed anxiety, and whether the addition of the child form for each measure significantly improved the predictive ability of either model. 

## 3. Results

### 3.1. Demographic Characteristics

The demographic information for the children and parents is presented in Table 1. 

#### 3.1.1. Descriptive Statistics and Comparison of Means

The scores on the SCQ ranged from 15 to 38 (M = 24.25, SD = 5.77), as those who scored below the threshold of 15 were excluded from the current study prior to analysis. 

The mean scores for the parents and children on the SCAS and the ASC-ASD are shown in Table 2. For the SCAS, the children rated their anxiety higher than their parents did on all but the Social Phobia and Physical Injury subscales. Similarly, the children also scored higher on the ASC-ASD total score, and the SA and U subscales compared to their parents, but not on the PA and AA subscales. However, a series of paired-samples *t*-tests indicated that the differences between the parent and child ratings were not significant for any subscale or total score (*p* > 0.05).

#### 3.1.2. Relationships between the Study Measures and Demographic Variables

Pearson correlation coefficients were calculated to assess whether the demographic variables related to the total scores of the ASC-ASD, SCAS, and SCQ. No significant relationships were observed between child age or gender and any of the study variables’ total scores. As all but three parents identified as female, the association between parent gender and the study variables was not examined. 

One-way ANOVAs were used to evaluate whether there was a relationship between the parent relationship status, employment status, educational level, geographical location, or family income bracket, and the total scores of the ASC-ASD, SCAS, and SCQ. In instances where the assumption of the homogeneity of variance was violated, Welch’s adjusted *F* ratio was used. 

There was a statistically significant difference in parent scores on the SCAS-P dependent upon the parent relationship status, *F*(3, 83) = 3.93, *p* = 0.011, with a medium effect size (eta squared = 0.12). Post hoc comparisons using the Tukey HSD test indicated that parents who were separated or divorced (M = 63.38, SD = 15.00) rated their child as significantly more anxious on the SCAS-P than parents who were living with a partner (M = 35.71, SD = 20.90). 

Similarly, scores on the ASC-ASD-P were also significantly different dependent upon the parental relationship status, *F*(3, 77) = 4.77, *p* = 0.004, with a large effect size (eta squared = 0.16). Post hoc comparisons using the Tukey HSD test revealed that the parents who were living together (M = 24.92, SD = 17.70) scored their child as less anxious on the ASC-ASD-P than the children of parents who were married (M = 37.00, SD = 13.16) or divorced or separated (M = 47.25, SD = 13.69).

While there were no significant differences observed based on the parent relationship status for the SCAS-C total, the scores did differ significantly on the ASC-ASD-C, Welch’s *F*(3, 20.52) = 6.41, *p* = 0.003, with a large effect size, ω^2^ = 0.22. Post hoc comparisons made with the Games–Howell Test indicated that the children of parents who were separated or divorced (M = 49.00, SD = 5.10) scored significantly higher on the ASC-ASD-C than the children of parents who were living together (M = 32.50, SD = 13.75) or married (M = 36.31, SD = 15.69). 

The scores on the SCAS-P and the ASC-ASD-P and -C did not significantly differ depending on the parent employment status. The differences in the scores on the SCAS-C, however, were statistically significant, *F*(4, 54) = 2.59, *p* = 0.047, with a large effect size (eta squared = 0.16). Although the pattern of results indicated that the children of parents with some form of paid employment (either part-time or full-time) had lower anxiety scores than the children of parents who were unemployed, students, or full-time parents, a post hoc analysis using the Tukey HSD test indicated that the differences between the groups were not significant. 

No significant differences between the total scores on the ASC-ASD, SCAS, or SCQ due to parent education level, geographical location, or family income bracket were observed. 

### 3.2. Clinically Anxious Versus Non-Anxious Children

As shown in Table 3, when comparing non-clinically anxious with clinically anxious children, the parents of anxious children rated their child higher on the SCAS-P and each of its subscales than the parents of those without an anxiety diagnosis. The independent samples *t*-tests indicated that these differences were significant for the total score, *t*(85) = 2.84, *p* = 0.006; Panic, *t*(85) = 2.52, *p* = 0.014; Social Phobia, *t*(85) = 3.14, *p* = 0.002; and Generalized Anxiety subscales, *t*(85) = 2.34, *p* = 0.022. The difference in scores on the Separation Anxiety subscale also approached significance, *t*(85) = 1.97, *p* = 0.052. The remaining differences were not significant (*p* > 0.05). 

For the SCAS-C, the children with clinical anxiety scored higher than those without an anxiety disorder diagnosis on both the total score and each of its subscales, also shown in Table 3. However, the independent samples *t*-tests indicated that the only significant difference in the scores was on the Generalized Anxiety subscale, *t*(57) = 2.06, *p* = 0.044.

The parents of clinically anxious children also rated their children higher on the ASC-ASD-P and each of its subscales than the parents of non-clinically anxious children, as shown in Table 4. This difference was significant for the total score, *t*(79) = 3.60, *p* = 0.001, and Performance Anxiety, *t*(76.03) = 3.39, *p* = 0.001, Anxious Arousal, *t*(79) = 2.72, *p* = 0.004, and Uncertainty subscales, *t*(79) = 3.09, *p* = 0.003. 

The scores for children with an anxiety disorder were also higher on the ASC-ASD-C compared to their non-anxious peers. However, none of these differences were significant (*p* < 0.05).

### 3.3. Correct Classification, Sensitivity, and Specificity of the Anxiety Measures

As shown in Table 5, of the 81 parents who completed both anxiety measures, 73 (90.1%) have a total *t*-score higher than 60 on the SCAS-P, and 69 (85.2%) have a total score greater than 20 on the ASC-ASD-P, indicating significant or elevated levels of anxiety. Although only 31 children had clinically diagnosed anxiety, both measures suggest that over twice that many children in this study have significant anxiety. Overall, 45.6% of the parents had their child’s anxiety disorder status correctly categorized by the SCAS-P, and 50.6% correctly classified by the ASC-ASD-P. 

As seen in Table 6, of the 56 children who completed both anxiety measures, 36 (64.3%) score above the threshold of the SCAS-C total score, and 44 (78.6%) score above the threshold of the ASC-ASD-C total score, signifying significant levels of anxiety. Fifty percent of the children had their anxiety disorder status correctly classified by the SCAS-C as well the ASC-ASD-C, although the distribution of true positives and true negatives differed. Specifically, the ASC-ASD-C had a higher rate of true positives, while the SCAS-C had a higher rate of true negatives. 

The sensitivity, specificity, and predictive values of the anxiety measures for the whole sample are shown in Table 7. While both the SCAS-P and ASC-ASD-P are observed to be highly sensitive, the ASC-ASD-P is more specific. The ASC-ASD-C is also more sensitive than the SCAS-C, but less specific. The positive predictive values (PPVs) do not vary much across the measures or informants, although both the parent and child forms of the ASC-ASD have higher PPVs compared to the appropriate version of the SCAS. 

### 3.4. Convergent and Divergent Validity

Pearson correlation coefficients were calculated to assess whether the SCQ was associated with either the total score or any of the subscale scores of the anxiety measures. The SCQ was significantly and positively correlated with three of the subscales, with weak associations observed with SCAS-P Panic, *r*(85) = 0.23, *p* = 0.034, and ASC-ASD-C Uncertainty, *r*(54) = 0.28, *p* = 0.04, and a moderate association observed with ASC-ASD-P Uncertainty, *r*(79) = 0.31, *p* = 0.005. The SCQ was not significantly correlated with any other anxiety score, indicating the relatively good divergent validity of the measures. 

The Pearson correlation coefficients between both the child and parent forms of the SCAS and the ASC-ASD are shown in Table 8. Overall, the ASC-ASD and SCAS display excellent convergent validity. Very strong, significant positive correlations are observed between the total scores of the SCAS-P and ASC-ASD-P, as well as between the SCAS-C and ASC-ASD-C, indicating that the children (or parents) who scored highly on one measure are likely to also score highly on the other. 

In addition, the overall pattern of results indicated that the children who rated themselves as more anxious also tended to be rated as more anxious by their parents. The SCAS-P and SCAS-C were strongly, significantly, and positively correlated, as were the parent and child forms for each subscale. Similarly, the parent and child scores for the ASC-ASD-P and ASC-ASD-C total score and all four subscales were strongly and significantly correlated, with each of these associations being positive in direction. The relationship between the parent forms of the measures and between the child forms of the measures was also stronger than between the parent and child forms of each measure. 

Strong correlations were also observed between the subscales proposed to measure the same ‘type’ of anxiety. Specifically, strong positive correlations were observed between the ASC-ASD-P and SCAS-P Separation Anxiety subscales, as well as the child forms of these subscales, both of which were significant.

### 3.5. Predictive Ability of the Anxiety Measures

Binomial sequential logistic regression analyses were performed to determine whether the SCAS or ASC-ASD was a significant predictor of parent-reported clinically diagnosed anxiety. The SCAS-P and ASC-ASD-P total scores were used as predictors for the initial models, with the child age and gender also included. The child total scores from the SCAS-C and ASC-ASD-C were added in Block 2 to evaluate whether the addition of the child form of each measure significantly improved the predictive ability of either model.

#### 3.5.1. Predicting Anxiety Status with the SCAS

The logistic regression model containing the SCAS-P, child age, and gender did not reach significance, χ^2^(3) = 7.20, *p* = 0.066, indicating that the model could not consistently distinguish between respondents who reported that their child did have an anxiety disorder versus those who reported no such disorder. The model, as a whole, correctly classified 71.2% of the cases and accounted for 15.4% of the variance in the data (Nagelkerke *R*^2^). As shown in Table 9, only the SCAS-P makes a unique significant contribution to the model. For every one-point increase on the SCAS-P, the children ae 1.04 times more likely to have an anxiety disorder. 

When the SCAS-C was added in Block 2, the model was still not significant, χ^2^(4) = 7.42, *p* = 0.116. A minimal increase was observed in the amount of variance explained by the model (15.9%; Nagelkerke *R*^2^), and the number of cases correctly identified decreased to 67.8%. 

#### 3.5.2. Predicting Anxiety Status with the ASC-ASD

The logistic regression model containing the ASC-ASD-P, child age, and gender was statistically significant, χ^2^(3) = 8.42, *p* = 0.038, indicating that the model could distinguish between the respondents who reported that their child did have an anxiety disorder versus those who reported no such disorder. Overall, the whole model correctly classified 64.3% of the cases and accounted for 18.7% of the variance in the data (Nagelkerke *R*^2^). As shown in Table 10, the ASC-ASD-P is the only predictor that makes a unique significant contribution to the model. For every one-point increase on the ASC-ASD-P, the children are 1.06 times more likely to have an anxiety disorder. 

When the ASC-ASD-C was added in Block 2, the model was still significant, χ^2^(4) = 9.68, *p* = 0.046, although it explained slightly less variance (21.3%; Nagelkerke *R*^2^). In addition, the number of cases correctly identified increased minimally to 67.9%. The ASC-ASD-P remains the only predictor that is a unique significant contributor to the model, as shown in Table 10. 

## 4. Discussion

The aim of this study was to extend on the findings of den Houting et al. [22] and assess the utility and validity of the ASC-ASD compared to the SCAS. As anticipated, the children with a parent-reported anxiety disorder had significantly higher ASC-ASD-P and SCAS-P total scores compared to their non-anxious counterparts. However, there was no significant difference in the total scores between the children with and without anxiety disorders for either the ASC-ASD-C or the SCAS-C, which was contrary to expectations. Similarly, more children had their anxiety disorder status correctly classified by the ASC-ASD-P than the SCAS-P, which was consistent with our predictions, although there was no difference in the percentage of children correctly classified by the child forms of each measure. Despite this, the PPV of both the child and parent forms of the ASC-ASD was higher than the corresponding version of the SCAS (i.e., 45.5% versus 44.4% for children, 43.5% versus 41.1% for parents). Overall, these findings suggest that the ASC-ASD may be more sensitive to detecting anxiety symptoms in autistic children in this age group compared to the SCAS, particularly when symptoms are rated by parents rather than the children themselves. 

It is of note that twice as many children scored over the cut-off points of the ASC-ASD-P (85.2%) and the SCAS-P (90.1%) than had a reported clinical diagnosis of anxiety (39.1%), indicating elevated levels of anxiety warranting further investigation amongst the majority of the children in this sample. While the percentage of children who self-rated their anxiety above the cut-off was less than the parent ratings (i.e., 78.6% for the ASC-ASD-C and 64.3% for the SCAS-C), it was still higher than the proportion that had a clinical diagnosis. The high incidence of anxiety issues within this study may reflect the tendency of parents of children with difficulties with anxiety to have self-selected to participate in this research, or the need for the further evaluation of the cut-off scores used on these self-report measures. Additionally, this finding may suggest that anxiety is underdiagnosed in this sample, potentially due to diagnostic overshadowing, where co-occurring mental health concerns, such as anxiety, are instead considered to be a part of the more prominent disability—in this case, autism characteristics [6]. As many of the children did in fact have a diagnosis, and there was a significant difference between the parent-rated anxiety scores for children with and without clinically recognised anxiety, this potentially suggests that only children with severe anxiety presentations are being diagnosed by health professionals, or even seeking a diagnosis in the first place. Considering that co-occurring anxiety in autistic children and adolescents has been associated with an increased risk of self-harm, symptoms of depression, and poorer quality of life [4,6], it is vital that appropriate methods for screening autistic individuals for the presence of even mild or moderate forms of anxiety are identified to ensure that all individuals who require support are receiving it, not only those who are experiencing severe symptoms. 

In line with our predictions, the results also demonstrated that the ASC-ASD had excellent convergent validity with the SCAS, and good divergent validity with the SCQ. Strong associations were observed between both the parent total scores and both child total scores, as well as between the corresponding total scores of the parent and child forms of the ASC-ASD and the SCAS. Conversely, the SCQ was not significantly associated with any of the total scores. This finding adds to the growing body of evidence for the validity of the ASC-ASD [17,19,33], and is consistent with the results of den Houting et al. [22], who observed moderate-to-strong correlations between the total scores for the parent forms of the ASC-ASD and the SCAS, as well as between the child forms for each measure. The correlations observed between the parent and child forms of the anxiety measures were much stronger in the present study than those observed in some of the previous research, e.g., [19,22,29,30], which tended to be weak to moderate. However, this result supports other findings that have demonstrated good agreement between parent and child anxiety ratings in autistic youth [25,34]. 

One possible reason for this stronger agreement between the parent and child ratings in the present research is due to the verbal skills of the children in the sample. Previous research by Blakeley-Smith et al. [24] found an association between verbal ability and several anxiety domains as assessed by the SCARED, specifically school avoidance, separation anxiety, and the measure’s total anxiety score. Supporting this, Ooi et al. [35] also found that higher verbal ability in autistic children was associated with better parent–child agreement on anxiety symptoms as measured by the SCAS. As verbal ability was not assessed in the current research, it is possible that the present study’s sample consisted of children with relatively strong verbal skills, enabling them to better disclose information about their internal symptoms to their parents. Future research could explore the association between verbal skills and parent–child agreement on anxiety symptoms using the ASC-ASD, as the agreement between the parent and child anxiety scores was stronger for this measure compared to the SCAS in the present study. Furthermore, this finding suggests that verbal ability and child introspection skills should be considered when using multi-informant reports of anxiety, both in research and in clinical settings. 

While the SCQ was not significantly related to any of the total scores as anticipated, weak significant correlations were observed with some of the subscales, particularly the Uncertainty subscales for both the child and parent forms of the ASC-ASD. Similarly, den Houting et al. [18] reported a weak significant relationship between the ASC-ASD-P’s Uncertainty subscale and the SCQ, suggesting that this may reflect items from the subscale also tapping into the core characteristics of autism. This is unsurprising, given that some autistic traits share similarities with symptoms of anxiety that reflect an intolerance for uncertainty, including restricted and repetitive behaviours, avoidance of unexpected events to increase the predictability of daily life, and inflexibility with change [36,37], as well as findings that have suggested that the intolerance of uncertainty may mediate the relationship between anxiety and autism characteristics [38].

Lastly, the results support the hypothesis that the ASC-ASD is a superior predictor of clinically diagnosed anxiety in autistic children compared to the SCAS. While the logistic regression model for the ASC-ASD-P was significant, the model for the SCAS-P was not. Overall, the regression model for the ASC-ASD-P correctly classified 64.3% of the cases, and was not improved with the addition of the child form of the model. A comprehensive search of the literature revealed no other studies that have examined the ability of the ASC-ASD to predict child clinical anxiety disorder status; therefore, the present findings are novel, extend upon the work of den Houting [22], and contribute new evidence for the validity and utility of this measure. It is also of interest that the child form did not significantly improve the predictive ability of the ASC-ASD. 

In total, these findings suggest that while the child form of the ASC-ASD may have value for identifying specific targets for treatment, for example, specific sources of distress or anxiety in a child’s life, the use of the parent form alone is sufficient to screen for anxiety or to identify the presence of elevated anxiety symptoms in autistic children aged 8 to 12 years old. As the ASC-ASD was designed specifically to capture both the typical and atypical symptoms of anxiety which may be experienced by autistic people [17], it is of no surprise that this measure emerged as a better predictor of parent-reported clinically diagnosed anxiety in autistic children compared to the SCAS, which was previously found to have a different underlying factor structure when used in autistic and non-autistic groups [39]. Similarly, this is consistent with other research that has argued that traditional measures, like the SCAS, do not always encompass the full expression of anxiety in autistic individuals and should be interpreted with caution [11,15]. 

Of note, the results of this research also found a significant relationship between parent marital status and increased child anxiety, as measured by the SCAS-P, ASC-ASD-P, and ASC-ASD-C. The higher rates of anxiety seen in the children of parents who are divorced or separated is consistent with the findings of a recent meta-analysis, which demonstrated that in non-autistic groups, parental divorce was significantly associated with an increased risk of poorer mental health outcomes for their children, including depression, anxiety, suicidal ideation, and risk of suicide [40]. Surprisingly, little research has explored the impact of parent divorce and family breakdown on the mental health of autistic children. 

Another limitation is that a large proportion of the children in this study scored above the threshold indicative of elevated levels of anxiety for the ASC-ASD and the SCAS. This may be due to parents who have children with noticeable symptoms of anxiety being more likely to participate, given their knowledge of the focus of this study. Conversely, those with children who do not frequently demonstrate noticeable symptoms of anxiety, or whose anxiety does not interfere with their daily life or the lives of their family members, may have been less inclined to participate. A sample with more diverse anxiety experiences may have increased the ability of the ASD-ASD regression model to predict anxiety status. Thus, future research could assess the validity and predictive ability of the ASC-ASD using such a sample. 

## 5. Conclusions

In conclusion, the results of the present study replicate and extend upon the findings of den Houting et al. [22], and suggest that the ASC-ASD is a valid measure for assessing the symptoms of anxiety in autistic children. Furthermore, the current results provide evidence that, for this age group, using the parent form of the ASC-ASD alone is sufficient to assess children for the presence of elevated anxiety symptoms. When compared to a ‘traditional’ anxiety measure designed for non-autistic populations, like the SCAS, only the ASC-ASD parent form was a significant predictor of parent-reported clinically diagnosed anxiety disorders in autistic children. Overall, this suggests that the ASC-ASD may better reflect the experience of anxiety in autistic children, as it captures both the typical and atypical symptoms of anxiety compared to the SCAS, which only covers typical anxiety. Given the evidence presented in this study for the utility of the ASC-ASD, future research could explore how child anxiety, as assessed by this measure, relates to other factors that significantly impact the lives of autistic children and their families, such as child school refusal and non-attendance, or family functioning and well-being. 

## Figures and Tables

**Table 1:** Child and parent demographic characteristics reported by parent (*n* = 87).

		*n*	%
Child demographics			
Child gender	Male	55	63.2%
	Female	32	36.8%
Other diagnoses	Anxiety Disorder	34	39.1%
	Depression	3	3.4%
	Sensory Processing Disorder	13	14.9%
	Attention-Deficit Disorder or Attention-Deficit/Hyperactivity Disorder	35	40.2%
	Intellectual Disability or Global Developmental Delay	12	13.8%
	Oppositional Defiant Disorder or Conduct Disorder	5	5.7%
School type	Mainstream	67	77.0%
	Autism Specific	3	3.4%
	Distance Education/Home Schooled	5	5.7%
	Special Education School	6	6.9%
	Support Unit in a Mainstream School	6	6.9%
Parent and family demographics			
Parent gender	Male	2	2.3%
	Female	84	96.6%
	Non-Binary	1	1.1%
Relationship to child	Biological Parent	79	90.8%
	Foster Parent	1	1.1%
	Guardian	3	3.4%
	Relative Responsible for Raising the Child	4	4.6%
Marital status	Single	18	20.7%
	Living Together	14	16.1%
	Married	47	54.0%
	Separated or Divorced	8	9.2%
Primary carer	Yes	84	96.6%
Employment status	Full-Time Employed	7	8.0%
	Part-Time Employed	35	40.2%
	Student	8	9.2%
	Full-Time Parent or Unemployed	37	42.5%
Income	USD 0–20,000	10	11.5%
	USD 20,001–50,000	17	19.5%
	USD 50,001–75,000	13	14.9%
	USD 75,001–100,000	11	12.6%
	USD 100,001–150,000	25	28.7%
	USD 150,001	11	12.6%
Geographic location	Metropolitan/Inner city	21	24.1%
	Urban/Outer Suburbs	31	35.6%
	Regional	29	33.3%
	Remote	31	6.9%

**Table 2:** The means, standard deviations, and range of scores for parent and child informants on the ASC-ASD and the SCAS.

	Parent			Child		
	M	SD	Range	M	SD	Range
ASC-ASD						
Total Score	35.43	14.69	0–72	36.14	15.73	9–70
Separation Anxiety (SA)	7.56	4.06	0–15	8.30	4.57	0–15
Uncertainty (U)	15.14	6.0	0–24	15.23	6.07	4–24
Performance Anxiety (PA)	8.57	4.75	0–15	8.34	4.98	0–15
Anxious Arousal (AA)	4.17	3.73	0–18	4.27	3.95	0–18
SCAS						
Total Score	48.98	19.81	4–107	50.22	20.78	9–100
Panic (P)	7.53	5.23	0–24	8.32	5.67	0–22
Separation Anxiety (SA)	9.85	4.65	0–18	9.95	5.03	0–18
Physical Injury Fears (PI)	7.26	3.36	1–14	7.07	3.20	1–13
Social Phobia (SP)	10.21	4.85	0–18	9.86	5.50	0–18
Obsessive Compulsive (OC)	5.64	3.21	0–15	6.24	3.84	0–18
Generalized Anxiety (GA)	8.48	4.10	1–18	8.78	4.01	2–18

Note: ASC-ASD-P, *n* = 81; SCAS-P, *n* = 87; ASC-ASD-C, *n* = 56; SCAS-C, *n* = 59.

**Table 3:** The mean scores and standard deviations on the SCAS-P and SCAS-C between children with and without an anxiety disorder diagnosis.

	Child w/ Anxiety Disorder	Child w/o Anxiety Disorder
SCAS-P		
Total Score	56.21 (18.47)	44.34 (19.40) *
Panic	9.24 (5.72)	6.43 (4.61) *
Separation Anxiety	11.06 (4.44)	9.08 (4.66)
Physical Injury Fears	7.71 (3.19)	6.98 (3.47)
Social Phobia	12.15 (3.78)	8.96 (5.08) *
Obsessive Compulsive	6.32 (3.01)	5.21 (3.29)
Generalized Anxiety	9.74 (4.00)	7.68 (4.00) *
SCAS-C		
Total Score	55.24 (23.11)	46.53 (18.37)
Panic	9.80 (6.29)	7.24 (4.99)
Separation Anxiety	10.96 (5.17)	9.21 (4.87)
Physical Injury Fears	7.68 (3.42)	6.62 (2.98)
Social Phobia	10.28 (5.53)	9.56 (5.54)
Obsessive Compulsive	6.52 (4.34)	6.03 (3.49)
Generalized Anxiety	10.00 (4.49)	7.88 (3.41) *

* *p* < 0.05. Note: SCAS-P with anxiety, *n* = 34; SCAS-P without anxiety, *n* = 53; SCAS-C with anxiety, *n* = 25; SCAS-C without anxiety, *n* = 34.

**Table 4:** Mean scores and standard deviations on ASC-ASD-P and ASC-ASD-C between children with and without anxiety disorder diagnosis.

	Child w/ Anxiety Disorder	Child w/o Anxiety Disorder
ASC-ASD-P		
Total Score	42.39 (13.30)	31.13 (13.95) **
Separation Anxiety	8.52 (4.41)	6.96 (3.74)
Uncertainty	17.61 (5.12)	13.60 (6.00) **
Performance Anxiety	10.58 (3.70)	7.32 (4.92) **
Anxious Arousal	5.69 (4.45)	3.24 (2.88) **
ASC-ASD-C		
Total Score	39.46 (16.71)	33.66 (14.74)
Separation Anxiety	8.50 (4.59)	8.16 (4.63)
Uncertainty	16.92 (5.67)	13.97 (6.14)
Performance Anxiety	8.79 (4.63)	8.00 (5.27)
Anxious Arousal	5.25 (5.07)	3.53 (2.71)

** *p* < 0.01. Note: ASC-ASD-P with anxiety, *n* = 31; ASC-ASD-P without anxiety, *n* = 50; ASC-ASD-C with anxiety, *n* = 24; ASC-ASD-C without anxiety, *n* = 32.

**Table 5:** Classification of parent anxiety scores on SCAS-P and ASC-ASD-P for children with and without reported clinical anxiety.

	SCAS-P		ASC-ASD-P	
	Anxiety	No Anxiety	Anxiety	No Anxiety
Positive	30	43	30	39
Negative	1	7	1	11

**Table 6:** Classification of parent anxiety scores on SCAS-C and ASC-ASD-C for children with and without reported clinical anxiety.

	SCAS-C		ASC-ASD-C	
	Anxiety	No Anxiety	Anxiety	No Anxiety
Positive	16	20	20	24
Negative	8	12	4	8

**Table 7:** Sensitivity, specificity, and predictive values of SCAS and ASC-ASD.

	Sensitivity (%)	Specificity (%)	PPV (%)	NPV (%)
SCAS-P	96.8	14.0	41.1	87.5
ASC-ASD-P	96.8	22.0	43.5	91.7
SCAS-C	66.7	37.5	44.4	60.0
ASC-ASD-C	83.3	25.0	45.5	66.7

Note: PPV = positive predictive value; NPV = negative predictive value.

**Table 8:** Correlations between SCAS-P, SCAS-C, ASC-ASD-P, and ASC-ASD-C total scores and subscales.

	1	2	3	4	5	6	7	8	9	10	11	12	13	14	15	16	17	18	19	20	21	22	23
1. SCAS-P Total	–																						
2. SCAS-P Panic	0.88 **																						
3. SCAS-P SA	0.83 **	0.65 **																					
4. SCAS-P PI	0.71 **	0.54 **	0.64 **																				
5. SCAS-P SP	0.73 **	0.50 **	0.53 **	0.44 **																			
6. SCAS-P OC	0.65 **	0.57 **	0.44 **	0.38 **	0.28 **																		
7. SCAS-P GA	0.82 **	0.76 **	0.56 **	0.37 **	0.55 **	0.49 **																	
8. SCAS-C Total	0.77 **	0.68 **	0.71 **	0.47 **	0.50 **	0.53 **	0.58 **																
9. SCAS-C Panic	0.66 **	0.74 **	0.56 **	0.27 *	0.36 **	0.50 **	0.54 **	0.87 **															
10. SCAS-C SA	0.71 **	0.54 **	0.86 **	0.49 **	0.40 **	0.42 **	0.49 **	0.79 **	0.61 **														
11. SCAS-C PI	0.51 **	0.37 **	0.52 **	0.75 **	0.21	0.37 **	0.21	0.63 **	0.40 **	0.50 **													
12. SCAS-C SP	0.54 **	0.40 **	0.43 **	0.29 *	0.63 **	0.17	0.44 **	0.73 **	0.49 **	0.48 **	0.40 **												
13. SCAS-C OC	0.38 **	0.33 *	0.27 *	0.23	0.17	0.64 **	0.20	0.61 **	0.54 **	0.33 **	0.37 **	0.23											
14. SCAS-C GA	0.63 **	0.64 **	0.54 **	0.21	0.41 **	0.36 **	0.64 **	0.87 **	0.83 **	0.66 **	0.38 **	0.61 **	0.42 **										
15. ASD-P Total	0.91 **	0.80 **	0.75 **	0.51 **	0.77 **	0.47 **	0.74 **	0.70 **	0.65 **	0.66 **	0.33 *	0.54 **	0.22	0.65 **									
16. ASD-P SA	0.80 **	0.66 **	0.86 **	0.49 **	0.49 **	0.49 **	0.62 **	0.69 **	0.57 **	0.81 **	0.41 **	0.44 **	0.22	0.58 **	0.80 **								
17. ASD-P U	0.78 **	0.68 **	0.67 **	0.52 **	0.64 **	0.46 **	0.54 **	0.55 **	0.55 **	0.56 **	0.30 *	0.34 **	0.18	0.48 **	0.89 **	0.65 **							
18. ASD-P PA	0.61 **	0.40 **	0.41 **	0.27 *	0.85 **	0.15	0.55 **	0.45 **	0.28 *	0.35 **	0.13	0.66 **	0.08	0.43 **	0.75 **	0.41 **	0.58 **						
19. ASD-P AA	0.68 **	0.84 **	0.42 **	0.31 **	0.38 **	0.39 **	0.69 **	0.55 **	0.69 **	0.41 **	0.21	0.26 *	0.25	0.61 **	0.70 **	0.48 **	0.49 **	0.32 **					
20. ASD-C Total	0.71 **	0.66 **	0.68 **	0.35 **	0.53 **	0.40 **	0.59 **	0.93 **	0.82 **	0.75 **	0.46 **	0.74 **	0.42 **	0.87 **	0.75 **	0.65 **	0.63 **	0.56 **	0.53 **				
21. ASD-C SA	0.65 **	0.52 **	0.73 **	0.33 *	0.43 **	0.43 **	0.54 **	0.77 **	0.63 **	0.89 **	0.37 **	0.46 **	0.39 **	0.68 **	0.66 **	0.78 **	0.53 **	0.41 **	0.37 **	0.79 **			
23. ASD-C PA	0.47 **	0.63 **	0.41 **	0.27	0.58 **	0.21	0.37 **	0.70 **	0.46 **	0.46 **	0.37 **	0.89 **	0.24	0.62 **	0.51 **	0.36 **	0.34 *	0.72 **	0.19	0.73 **	0.50 **	0.61 **	
24. ASD-C AA	0.51 **	0.67 **	0.36 **	0.13	0.24	0.31 *	0.52 **	0.74 **	0.88 **	0.40 **	0.28 *	0.43 **	0.43 **	0.80 **	0.46 **	0.37 **	0.29 *	0.17	0.72 **	0.70 **	0.44 **	0.52 **	0.39 **

* *p* <.05 ** *p* <.001 Note: SA = Separation Anxiety, PI = Physical Injury Fears, SP = Social Phobia, OC = Obsessive Compulsive, GA = Generalized Anxiety, U = Uncertainty, PA = Performance Anxiety, AA = Anxious Arousal, SCAS = Spence Children’s Anxiety Scale, ASD = Anxiety Scale for Children with Autism Spectrum Disorder.

**Table 9:** Sequential logistic regression predicting likelihood of parent-reported child anxiety disorder from SCAS.

						Odds	95% CI For Odds Ratio	
	*B*	*SE*	Wald	*df*	*p*	Ratio	Lower	Upper
Step 1								
Child age	0.24	0.23	1.09	1	0.296	1.27	0.81	1.98
Child gender	0.41	0.58	0.51	1	0.477	1.51	0.49	4.65
SCAS-P	0.04	0.02	5.22	1	0.022	1.04	1.01	1.07
Constant	−4.56	2.40	3.60	1	0.058	0.01		
Step 2								
Child age	0.25	0.23	1.14	1	0.285	1.28	0.82	2.00
Child gender	0.46	0.59	0.61	1	0.435	1.58	0.50	5.01
SCAS-P	0.04	0.02	3.50	1	0.061	1.05	1.0	1.09
SCAS-C	−0.01	0.02	0.21	1	0.647	0.99	0.95	1.03
Constant	−4.56	2.41	3.58	1	0.059	0.01		

**Table 10:** Sequential logistic regression predicting likelihood of parent-reported child anxiety disorder from ASC-ASD.

						Odds	95% CI For Odds Ratio	
	*B*	*SE*	Wald	*df*	*p*	Ratio	Lower	Upper
Step 1								
Child age	0.27	0.24	1.34	1	0.247	1.31	0.83	2.08
Child gender	0.24	0.60	0.16	1	0.689	1.27	0.39	4.16
ASC-ASD-P	0.05	0.02	5.97	1	0.015	1.06	1.01	1.10
Constant	−4.96	2.50	4.07	1	0.044	0.01		
Step 2								
Child age	0.31	0.24	1.64	1	0.200	1.36	0.85	2.18
Child gender	0.35	0.62	0.32	1	0.572	1.42	0.42	4.81
ASC-ASD-P	0.08	0.03	5.62	1	0.018	1.08	1.01	1.16
ASC-ASD-C	-0.03	0.03	1.20	1	0.274	0.97	0.91	1.03
Constant	−5.20	2.50	4.33	1	0.038	0.01		

## Data Availability

The data that support the findings of this study are not openly available, but are available from the corresponding author upon reasonable request.
